# Amino acid-assisted green synthesis of a porous binary oxide nanocomposite for sensitive electrochemical detection of chlorpromazine

**DOI:** 10.1038/s41598-025-19185-2

**Published:** 2025-10-08

**Authors:** Sahar Zinatloo-Ajabshir, Abbas Pardakhty, Hadi Mahmoudi-Moghaddam, Hamid Akbari Javar

**Affiliations:** 1 Department of Chemical Engineering, University of Bonab, P.O. Box. 5551395133, Bonab, Iran; 2 Pharmaceutics Research Center, Institute of Neuropharmacology, Kerman University of Medical Sciences, Kerman, Iran; 3 Department of Pharmaceutics, Faculty of Pharmacy, Tehran University of Medical Sciences, Tehran, Iran

**Keywords:** Chlorpromazine, Nanocomposite, Modified electrode, Eco-friendly, Medical research, Analytical chemistry, Biochemistry, Electrochemistry, Green chemistry

## Abstract

In this work, an eco-friendly method was used to synthesize cerium–bismuth oxide nanostructures as a binary oxide nanocomposite. For this purpose, L-alanine was utilized as a novel and green fuel to produce a nanostructured Bi_2_O_3_–CeO_2_ sample. The prepared porous CeO_2_–Bi_2_O_3_ NPs and ionic liquid were used to develop a new sensor (Pr–CeO_2_–Bi_2_O_3_/IL/CPE) for detecting Chlorpromazine (CLP). The study showed that the present electrode has good electrochemical performance for CLP detection under a diffusion-controlled procedure. The chronoamperometric method was utilized to calculate the diffusion coefficient (D) of CLP, which was found to be 1.47 × 10^–5^ cm^2^ s^− 1^. The DPV technique was employed to establish the calibration curve, demonstrating a linear range of 0.02–140 µM and achieving a detection limit (LOD) of 9 nM. The prepared sensor also showed suitable repeatability and reproducibility. The practical applicability of Pr–CeO_2_–Bi_2_O_3_/IL/CPE for detecting CLP in various real samples showed good sensitivity and selectivity.

## Introduction

Chlorpromazine (CLP), a phenothiazine derivative with an aliphatic side chain, is commonly used to treat psychotic disorders. It helps manage agitation, excitement, and other psychomotor issues in individuals with schizophrenia and alleviates manic episodes in bipolar disorder^1,2^. CLP is also effective in controlling hyperactivity and aggressive behavior and is sometimes prescribed for anxiety and tension in various psychiatric conditions. Additionally, it serves as an antiemetic in palliative care^3,4^. Nevertheless, caution is necessary when administering CLP, as it can cause significant adverse effects in humans, such as endocrine imbalances, drowsiness, respiratory depression, blurred vision, episodic hypotension, dry mouth, constipation, nausea, akathisia, sleep disturbances, and dystonia^5,6^. Various analytical methods have been utilized to detect CLP, including capillary zone electrophoresis^7^, chemiluminescence^8^, HPLC^9^, and GC/MS^10^. Though these traditional techniques often require complex sample preparation, are costly, and time-consuming^11–13^. Recently, electrochemical detection methods have gained significant attention for their simplicity, affordability, rapid response, low detection limits, and excellent sensitivity and selectivity^14–18^. Nanomaterials are recognized for enhancing efficiency due to their morphology, surface properties, crystallinity, and particle size. Studies highlight that some materials, such as metals, metal oxides, rare earth oxides, graphene, carbon nanotubes, and fullerenes, are widely used in electrochemical sensing and biosensing applications^19–24^.

Metal oxide-based electrodes, in particular, offer a unique combination of features, including excellent electrical conductivity, large active surface area, and lightweight nature, making them highly promising for high-performance applications^25,26^. Bismuth oxide-based nanoparticles have emerged as promising electrode modifiers in electrochemical devices, due to their exceptional electron mobility^27^. Bi_2_O_3_ is known for its versatility and wide range of applications, attributed to its small energy band gap, high surface area, good conductivity, and photoconductive properties^28^. Additionally, Bi_2_O_3_ is an environmentally friendly material, serving as an excellent alternative to mercury in electrochemical analysis^29^.

Rare earth oxides have been widely studied in recent decades for their advanced applications in catalysis, optics, and electronics, largely due to unique features linked to the 4f electron shell^30^. Among them, cerium oxide (CeO_2_) has drawn considerable attention for its low cost, exceptional oxygen storage capacity, and high catalytic activity^31^. These properties make CeO_2_ highly suitable for a wide range of technological applications, such as catalysts, oxygen sensors, and fuel cells, owing to its chemical stability, high mechanical strength, and distinctive optical characteristics. With its redox capabilities and 4f shell availability, CeO_2_ shows significant potential as a catalyst in electrochemical devices^32,33^. Combining Bi_2_O_3_ with cerium dioxide (CeO_2_) nanoparticles as an electrode modifier offers a promising strategy to enhance the electrochemical performance of sensors^34^. This combination leverages the high electron mobility and electrocatalytic properties of Bi_2_O_3_, along with the excellent oxygen storage capacity and redox capabilities of CeO_2_. Together, they can significantly improve the electrode’s electrical conductivity and electrocatalytic activity, making it more effective for various sensing applications^35^.

Room-temperature ionic liquids (ILs) are a distinct class of non-molecular ionic solvents characterized by their low melting points. In recent years, they have gained significant prominence in materials science, biophysical chemistry, electrochemistry, and green reaction media^36,37^. This growing interest is due to their unique properties, such as their ability to interact with a wide range of chemicals, thermal stability, low toxicity, broad electrochemical window, and high ionic conductivity at room temperature, making them versatile for numerous applications^38^. 1-Butylpyridinium hexafluorophosphate (BPPF_6_) is an effective ionic liquid used in the fabrication of modified electrodes, demonstrating excellent performance as a working electrode material^39^.

Compared to traditional chemical methods, green synthesis uses eco-friendly and natural materials, including fruit peels, leaf extracts, bio-compounds, and microorganisms, as reducing substances. These green materials can also function as dispersants and end-capping agents, eliminating the need for harmful chemicals and minimizing energy consumption, thereby promoting sustainable and eco-friendly practices^40^.

This experimental study represents a facile and efficient green route for the fabrication of cerium bismuth oxide-based nanostructures by employing L-alanine molecules as novel and eco-friendly fuel and morphology guiding agents, as well as metal nitrates. L-alanine molecules are biodegradable and non-harmful, and owing to their functional groups and bulky structure, they can simultaneously act as a fuel and a morphology directing agent, and have a significant positive effect in achieving cerium bismuth oxide-based nanostructure. In addition, a sensitive sensor was developed using a CPE modified with an ionic liquid and Pr–CeO_2_–Bi_2_O_3_ for the electrochemical determination of CLP. Electrochemical analysis results demonstrated that the modified electrode exhibited excellent sensitivity, making it highly effective for CLP detection.

## Experimental

### Chemicals and reagents

N-butyl pyridinium hexafluorophosphate (BPPF6, 97%), monobasic and dibasic sodium phosphates (99%), KCl (98%), sodium hydroxide (99%), K_4_[FeCN_6_] and K_3_[FeCN_6_] (99%), were purchased from Merck Company. Bismuth (III) nitrate pentahydrate (99.5%), L-alanine (analytical grade), Cerium (III) nitrate hexahydrate (99%), and Chlorpromazine hydrochloride (99%) were from Sigma-Aldrich.

Electrochemical measurements in this study were conducted using an Autolab potentiostat/galvanostat (PGSTAT204). The experiments were performed in a three-electrode electrochemical setup. The microscopic morphology of the as-produced cerium bismuth oxide-based nanostructure sample was evaluated with FESEM (Zeiss Sigma 300) and TEM (Philips CM30). Identification of the nature of crystallinity and phase of the synthesized cerium bismuth oxide-based sample was conducted employing a Philips X-ray diffractometer. Further, the elemental composition of the as-prepared cerium bismuth oxide-based sample was analyzed using EDS.

### An eco-friendly method for the synthesis of cerium bismuth oxide-based nanostructure

A simple, eco-friendly approach was introduced to synthesize cerium bismuth oxide-based nanostructures. L-alanine molecules were applied as fuel and morphology directing agents for the first time. Initially, 1 mmol of Ce(NO_3_)_3_.6H_2_O and 0.4 mmol of Bi(NO_3_)_3_·5H_2_O were introduced into 15 mL of deionized water and stirred. Subsequently, 7 mL of L-alanine solution was gradually added to the solution, dropwise, comprising metal nitrates. The cerium to alanine molar ratio was maintained at 1:5. Next, the resulting product was stirred at 45 °C for half an hour. Then, the temperature was increased to about 120 °C to obtain a gel-like product. After that, the precipitate was dried at 75 °C for a duration of 12 h, after calcination at 600 °C for 100 min, and finally, the cerium bismuth oxide-based nanostructure was obtained.

### Preparation of carbon paste electrodes (CPE)

The Pr–CeO_2_–Bi_2_O_3_/IL/CPE was fabricated by manually blending 0.45 g of powdered graphite, 50 mg of Pr–CeO_2_–Bi_2_O_3_ NPs, 0.4 mL of BPPF_6_, and 0.8 mL of mineral oil. The resulting homogeneous mixture was then packed into a cylindrical plastic tube, with a copper wire inserted to ensure electrical contact with the external circuit. Before use, deionized water was applied to rinse the electrode surface.

The IL/CPE and bare CPE were prepared using the same method, with the only difference being the exclusion of Pr–CeO_2_–Bi_2_O_3_ and/or BPPF_6_, as appropriate. By keeping the amount of graphite and mineral oil constant across all formulations, we ensured that the physical properties of the paste (e.g., viscosity and conductivity) remained consistent, allowing for reliable comparison between electrodes.

### Real sample analysis

A pharmaceutical tablet containing 100 mg of chlorpromazine (Tehran Chemie Pharmaceutical Company, Iran) was purchased from a local pharmacy. The tablet was finely ground using a mortar and pestle. An accurately weighed portion of the resulting powder was placed in a 100 mL volumetric flask and dissolved in 0.1 M PBS. The obtained solution was subsequently sonicated for 5 min to ensure complete dissolution. Finally, the electrochemical analysis of chlorpromazine (CLP) was performed using the standard addition method.

To analyze urine samples, they were spiked with different concentrations of chlorpromazine (CLP). These were subsequently diluted tenfold with PBS, and the pH of the final solution was set to 7.0.

## Results and discussion

### Characterization of Pr–Bi_2_O_3_–CeO_2_ NPs

**Fig. 1:**
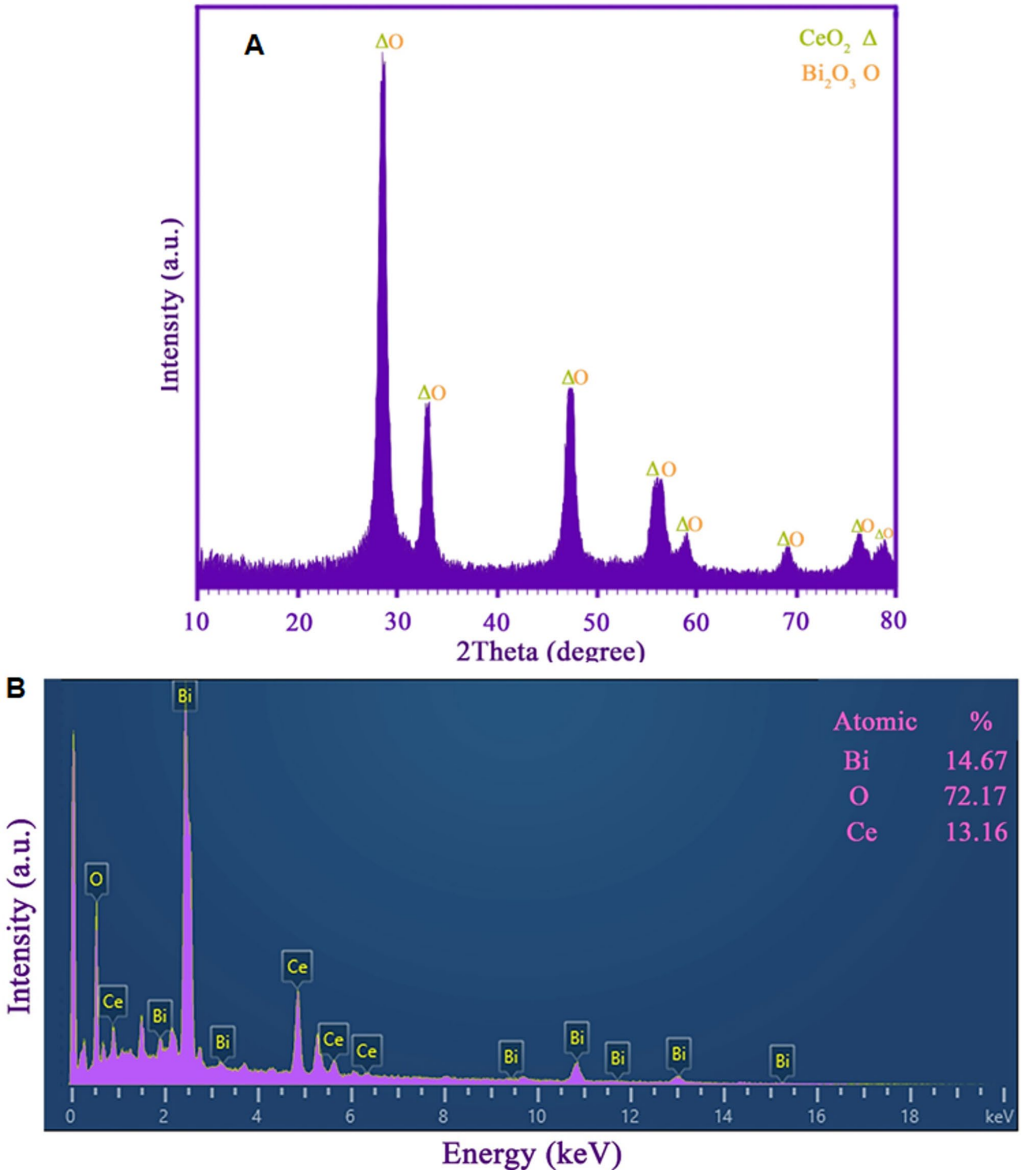
(**A**) XRD data and (**B**) EDS spectrum of the cerium bismuth oxide-based nanostructure produced using L-alanine molecules by an eco-friendly approach.

XRD data of the synthesized sample in the presence of L-alanine molecules are depicted in Fig. 1A. The XRD pattern exhibits peaks attributed to CeO₂ (JCPDS card 01-075-0076) as well as Bi_2_O_3_ (JCPDS no. 01-076-2478) with cubic phase^41^. No secondary signal appeared in the XRD pattern, demonstrating the purity of the fabricated sample in the presence of L-alanine molecules. Furthermore, with the presence of strong signals, it seems that the as-produced oxide sample has good crystallinity. The crystal size of the as-synthesized cerium bismuth oxide-based sample was calculated to be near 15 nm employing the Scherrer equation^42^. By examining the results of EDS analysis, which was carried out to examine the chemical composition of the product in the presence of L-alanine (see Fig. 1B), it is apparent that only signals related to cerium, oxygen, and bismuth appeared. The findings are consistent with the XRD results, confirming the purity of the sample.

**Fig. 2:**
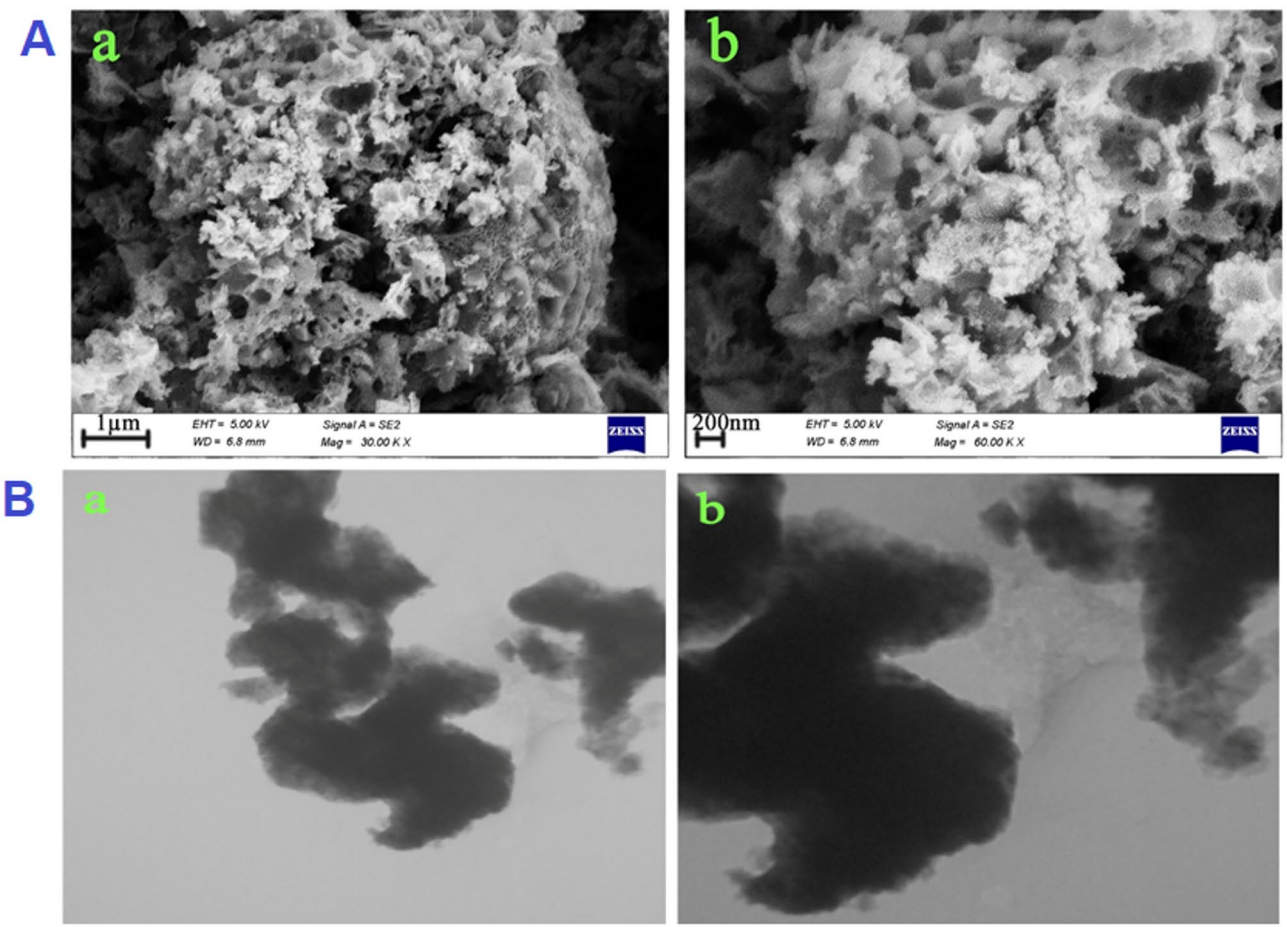
(**A**) SEM and (**B**) TEM images of the cerium bismuth oxide-based nanostructure at different magnifications.

The assessment of the microstructure and surface morphological characteristics of the Pr–Bi_2_O_3_–CeO_2_ NPs was carried out using FESEM, and the resulting images are presented in Fig. 2A. Images taken from the sample clearly illustrate the development of a porous nanostructure in the sample. Since L-alanine molecules have a bulky structure and functional groups, they seem to simultaneously play a remarkable positive role as both a fuel and a morphology-guiding agent in forming a porous-like nanostructure^43–45^.

TEM images recorded from the as-synthesized cerium bismuth oxide-based sample with the aim of examining its interior morphology are depicted in Fig. 2B. The images of the oxide sample reveal a porous nanostructure created by the assembly of spherical and smaller nanoparticles, confirming the FESEM findings.

### Electrochemical behavior of Pr–CeO_2_–Bi_2_O_3_/IL/CPE

**Fig. 3:**
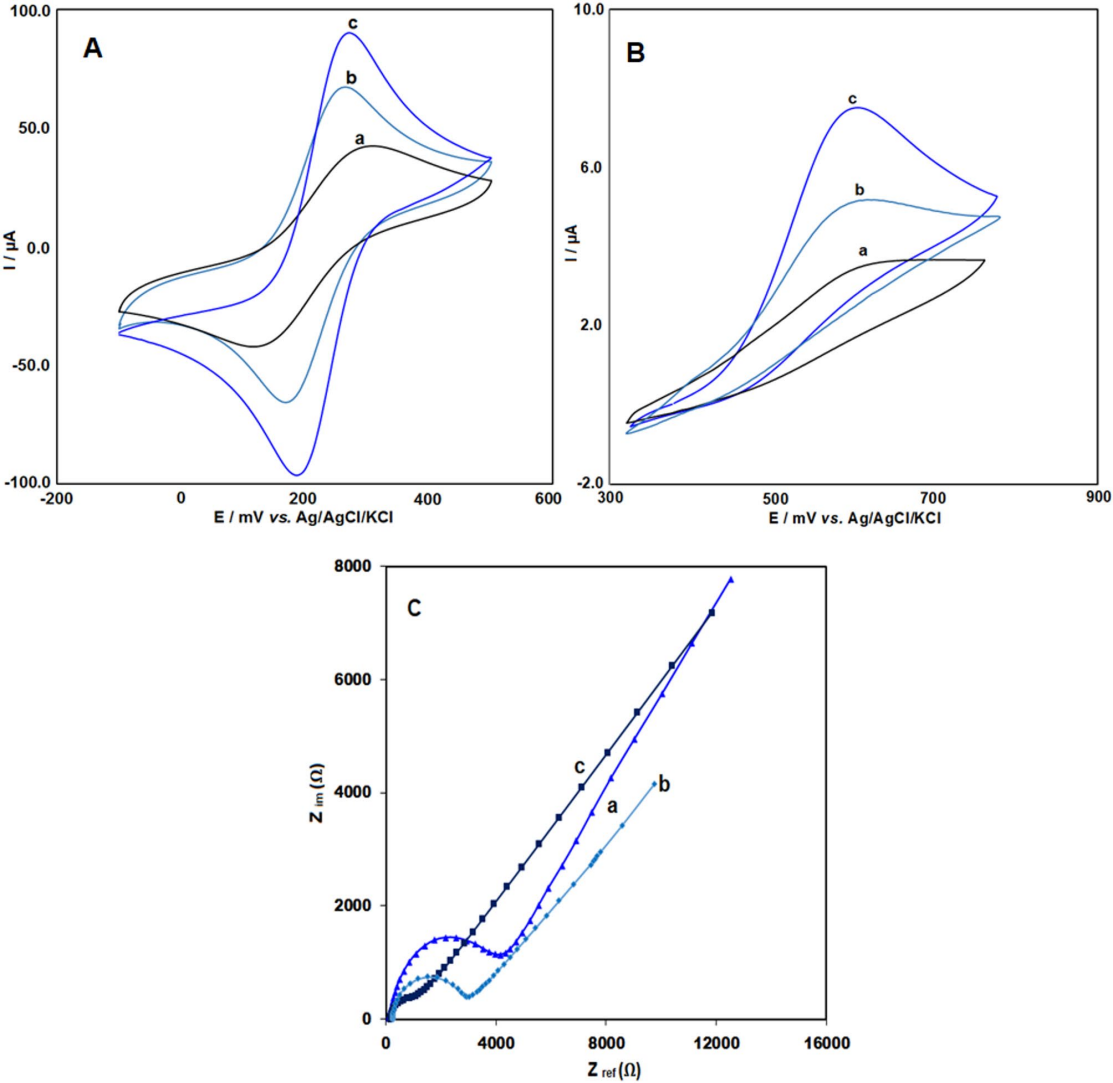
Cyclic voltammetry (CV) of (**a**) bare CPE, (**b**) IL/CPE, and (**c**) Pr–CeO_2_–Bi_2_O_3_/IL/CPE in (A) a 5 mM solution of [Fe(CN)_6_]^3−^/^4−^ in 0.5 M ABS (pH = 4.8) and (B) 50 µM of CLP in 0.1 M PBS (pH = 7); scan rate 50 mV/s and (C) Nyquist plots of the impedance spectra recorded in 0.5 mM [Fe(CN)_6_]^3−^/^4−^ containing 0.1 M KCl.

The electrochemical properties of the Pr–CeO_2_–Bi_2_O_3_/IL/CPE electrode were evaluated using the well-established redox reaction of potassium ferricyanide (K_3_[Fe(CN)_6_]). Figure 3A illustrates the cyclic voltammograms of the bare CPE (a), IL/CPE (b), and Pr–CeO_2_–Bi_2_O_3_/IL/CPE (c) in the presence of a 5 mM [Fe(CN)_6_]^3−/4−^ solution with 0.1 M KCl. The bare CPE displayed a pair of distinct redox peaks with a weaker redox peak current than the other electrodes. When modified with ionic liquid (IL), the redox peak current is enhanced, which can be ascribed to the improved conductivity provided by the ionic liquid (IL) on the electrode surface. Notably, the Pr–CeO_2_–Bi_2_O_3_/IL/CPE demonstrated a significantly higher redox signal and a narrower peak-to-peak separation (ΔE_p_) compared to the other electrodes. This improvement is due to the synergistic effect of the ionic liquid’s conductivity and the excellent electrochemical performance of Pr–CeO_2_–Bi_2_O_3_.

The electrochemically active surface area of both the Pr–CeO_2_–Bi_2_O_3_/IL/CPE and bare CPE was determined using Eq. (1)^23^:

1 $${\text{I}}_{{{\text{pa}}}} ~ = ~{\text{2}}.{\text{69}}~ \times ~{\text{1}}0^{{\text{5}}} {\text{n}}^{{{\text{3}}/{\text{2}}}} {\text{A C}}_{{\text{o}}} {\text{D}}^{\raise.5ex\hbox{$\scriptstyle 1$}\kern-.1em/ \kern-.15em\lower.25ex\hbox{$\scriptstyle 2$} } v^{\raise.5ex\hbox{$\scriptstyle 1$}\kern-.1em/ \kern-.15em\lower.25ex\hbox{$\scriptstyle 2$} }$$

The calculated effective surface areas were 0.052 cm^2^ for the bare CPE and 0.12 cm^2^ for the Pr–CeO_2_–Bi_2_O_3_/IL/CPE, indicating a significant increase in surface area due to the modification.

Figure 3B shows the cyclic voltammetry (CV) curves for 50 µM chlorpromazine (CLP) at (a) the bare CPE, (b) IL/CPE, and (c) Pr–CeO_2_–Bi_2_O_3_/IL/CPE. All electrodes exhibited an oxidation peak for CLP, but with significant differences in peak current. The bare CPE displayed a low oxidation peak current, indicating limited electron transfer as a result of low conductivity. Following the modification of the electrode with an ionic liquid (IL/CPE), the peak current increased, demonstrating enhanced conductivity and improved electron transfer. The highest oxidation peak current was observed with the Pr–CeO_2_–Bi_2_O_3_/IL/CPE. The observed improvement can be ascribed to the synergistic effects of suitable conductivity of the ionic liquid, the appropriate electrochemical performance of Pr–CeO_2_–Bi_2_O_3_, and the large surface area of the modified electrode. This combination significantly enhanced the overall electrochemical efficiency, making the modified electrode highly effective for CLP detection.

Figure 3C shows the Nyquist plots of the electrochemical impedance spectra recorded in 0.5 mM [Fe(CN)_6_]^3−^/^4−^ containing 0.1 M KCl for (a) CPE, (b) IL/CPE, and (c) CeO_2_–Bi_2​_O_3_​/IL/CPE.

The bare CPE (curve a) exhibits the largest semicircle, indicating the highest charge-transfer resistance due to its limited electron transfer capability (Rct = 3950 Ω). In contrast, IL/CPE (curve b) shows a smaller semicircle, reflecting a decrease in Rct (Rct = 2650 Ω)​. This improvement arises from the presence of the ionic liquid, which provides high ionic conductivity, a more favorable microenvironment, and better electrode–electrolyte interfacial contact, thereby facilitating faster charge transfer. Among the tested electrodes, CeO_2_–Bi_2_O_3_​​/IL/CPE (curve c) displays the smallest semicircle diameter, corresponding to the lowest Rct​ and the most efficient electron transfer kinetics (Rct = 850 Ω). This result demonstrates the synergistic effect of CeO_2_​–Bi_2_O_3​_ nanomaterials and the ionic liquid in enhancing the electrochemical performance of the electrode.

### Effect of pH on CLP oxidation

**Fig. 4:**
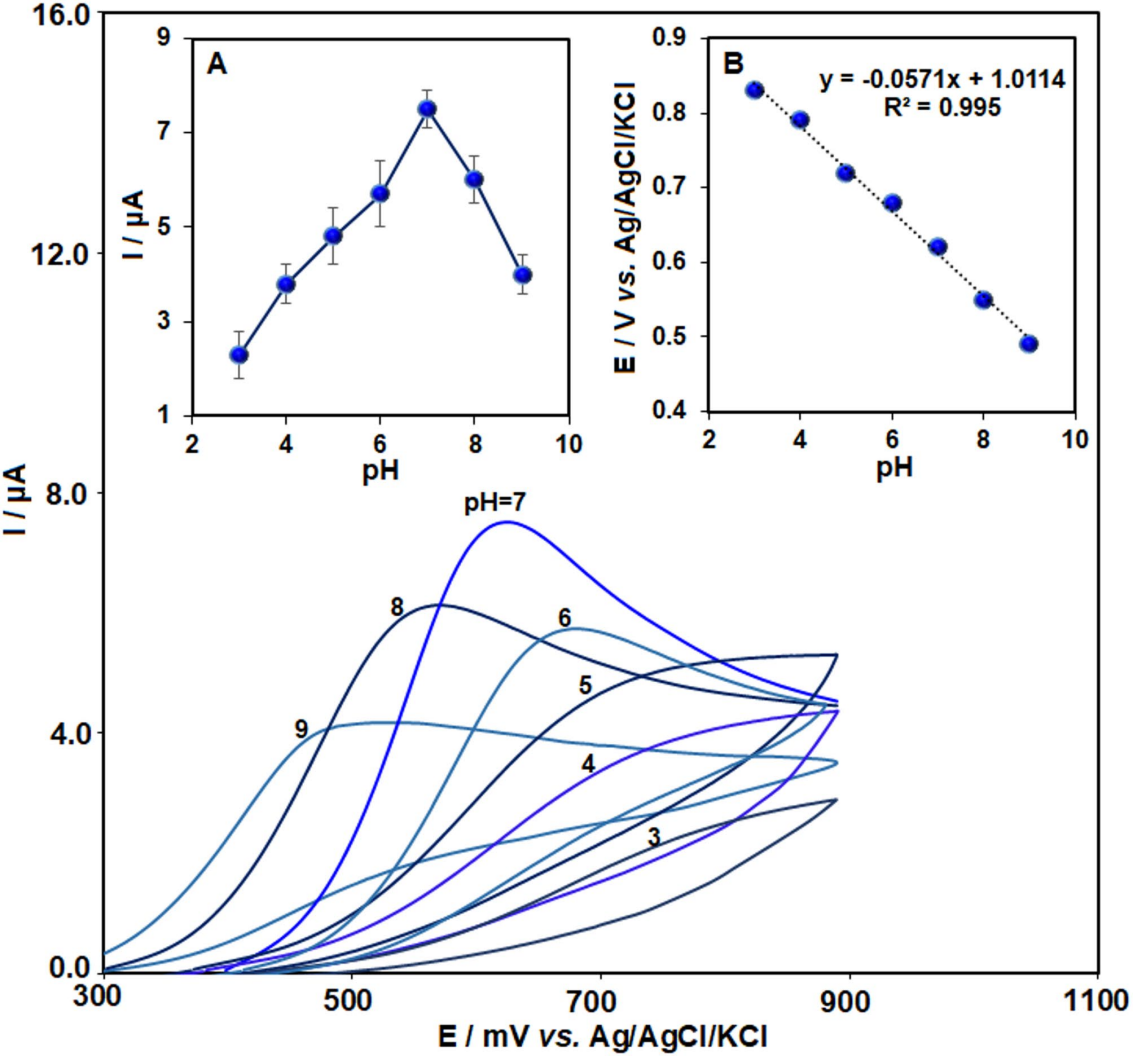
Cyclic voltammograms of Pr-CeO₂-Bi₂O₃/IL/CPE in the presence of 50 µM chlorpromazine (CLP) in 0.1 M PBS solution at various pH values (3.0–9.0). Insets: (**A**) The graph of oxidation peak current versus pH values (*n* = 3); (**B**) The graph of peak potential versus pH values.

Cyclic voltammetry was employed to investigate the impact of solution pH on the oxidation of chlorpromazine (CLP) in a 0.1 M PBS. As shown in Fig. 4, for 50 µM CLP, the peak current exhibited a gradual increase with the solution pH rising from 3 to 7, reaching its maximum at neutral pH. Beyond pH 7, the oxidation peak current markedly decreased, suggesting that CLP electro-oxidation is most efficient in a neutral medium. Consequently, pH 7 was chosen for subsequent experiments (Fig. 4-inset A).

Inset B of Fig. 4 shows that the oxidation peak potential shifted toward more negative potentials with rising, suggesting that protons are directly involved in the oxidation process. This relationship follows the equation of E_p.a._ (V)= − 0.0571 (pH) + 1.0114. A slope of 0.057 V/pH, close to the theoretical value of 59 mV, suggests that an equal number of protons and electrons are involved in the reaction. Therefore, based on the predicted mechanism, the oxidation of CLP on the Pr–CeO_2_–Bi_2_O_3_/IL/CPE electrode involves the transfer of one electron and one proton.

**Fig. 5:**
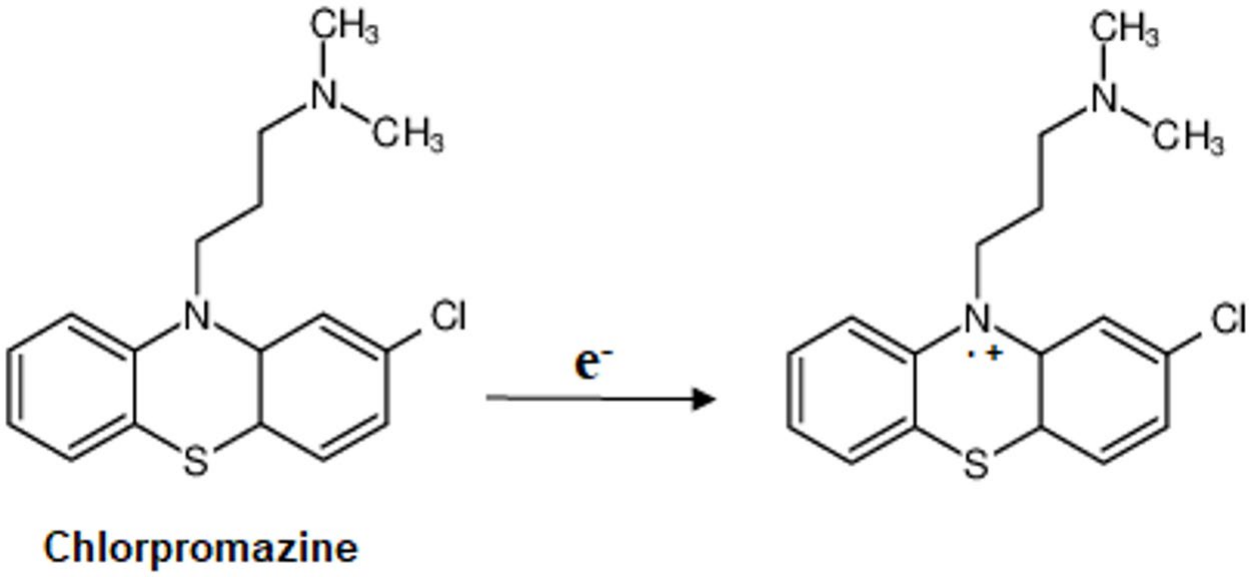
Electrochemical mechanism for CLP oxidation.

The electrochemical oxidation of chlorpromazine (CLP) involves the transfer of one electron from the nitrogen atom in the phenothiazine ring, which leads to the generation of a radical cation, as shown in Fig. 5. At neutral pH (pH 7), the protonation state of CLP is optimal for efficient electron transfer. Under acidic conditions, excessive protonation can stabilize the lone pair on the nitrogen atom, thereby increasing the oxidation potential and reducing the oxidation current. In contrast, in basic media, deprotonation can modify the electronic structure of the molecule, diminishing the availability of the nitrogen’s lone pair for oxidation and thus lowering the electron transfer rate. Furthermore, the radical cation formed during the oxidation of CLP is more stable at neutral pH. This stability helps to suppress unwanted side reactions such as hydrolysis or disproportionation, which are more likely to occur under strongly acidic or alkaline conditions. Consequently, pH 7 provides the most favorable environment for the electrochemical detection of CLP, ensuring both a higher signal intensity and better repeatability.

### Scan rate study

**Fig. 6:**
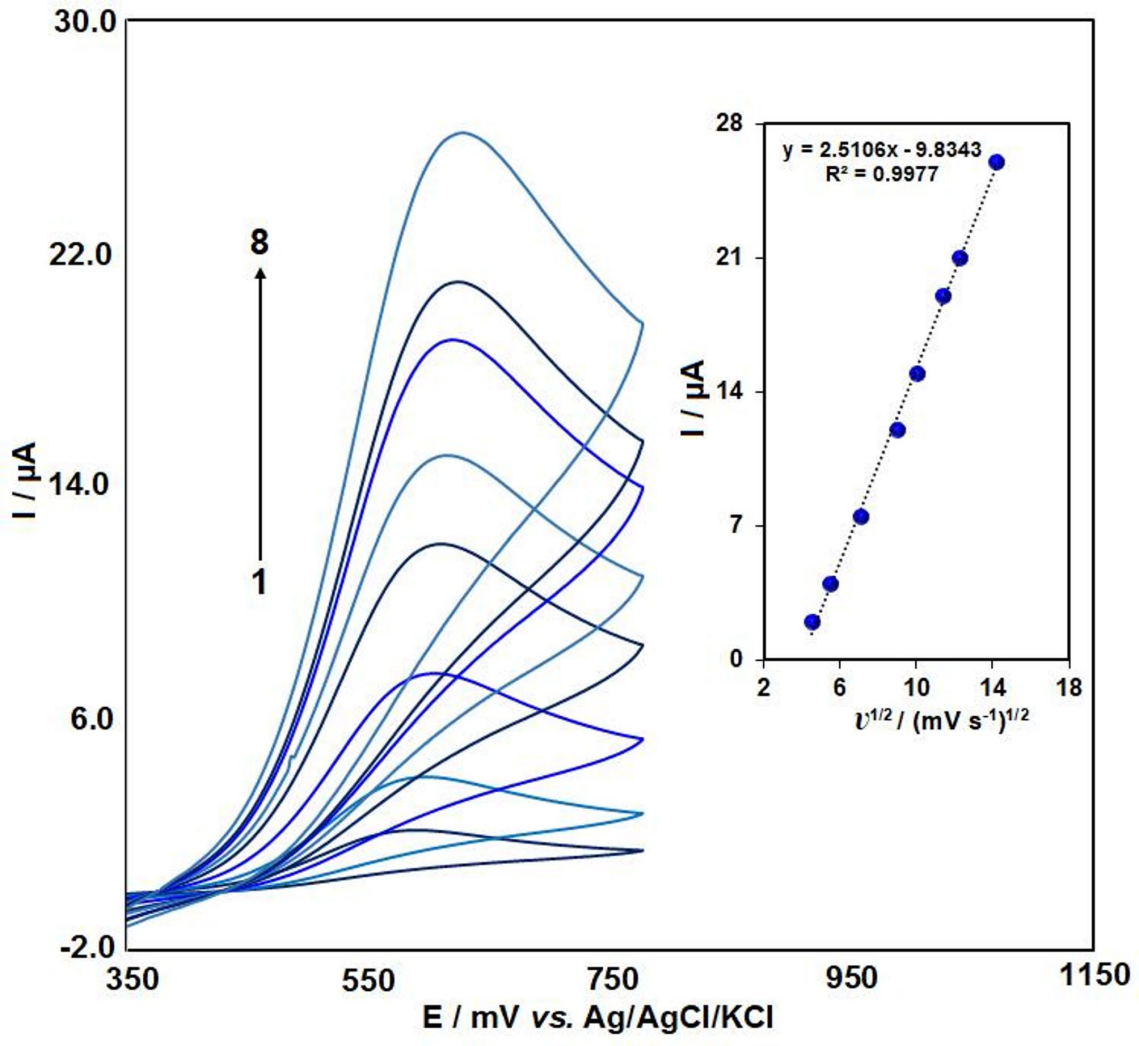
Cyclic voltammetry of 50 µM CLP on Pr–CeO_2_–Bi_2_O_3_/IL/CPE in PBS (0.1 M, pH 7) with scan rates between 30 and 250 mV/s (1 to 8, respectively). Inset: The changes in the peak current against the scan rate square root.

Figure 6 presents the CV response of CLP on the Pr–CeO_2_–Bi_2_O_3_/IL/CPE over a range of scan rates. The peak current exhibited a progressive increase with the scan rate over the range of 30–250 mV/s. The inset in Fig. 6 illustrates the correlation between the I_p_ and *v*^1/2^. The signals of CLP showed a linear correlation with the scan rate square root, with the equation I_p.a._ (µA) = 2.5106 *v*^1/2^ (mV/s) − 9.8343. This behavior indicates that the oxidation of CLP on the modified CPE follows a typical diffusion-controlled process.

### Chronoamperometry

**Fig. 7:**
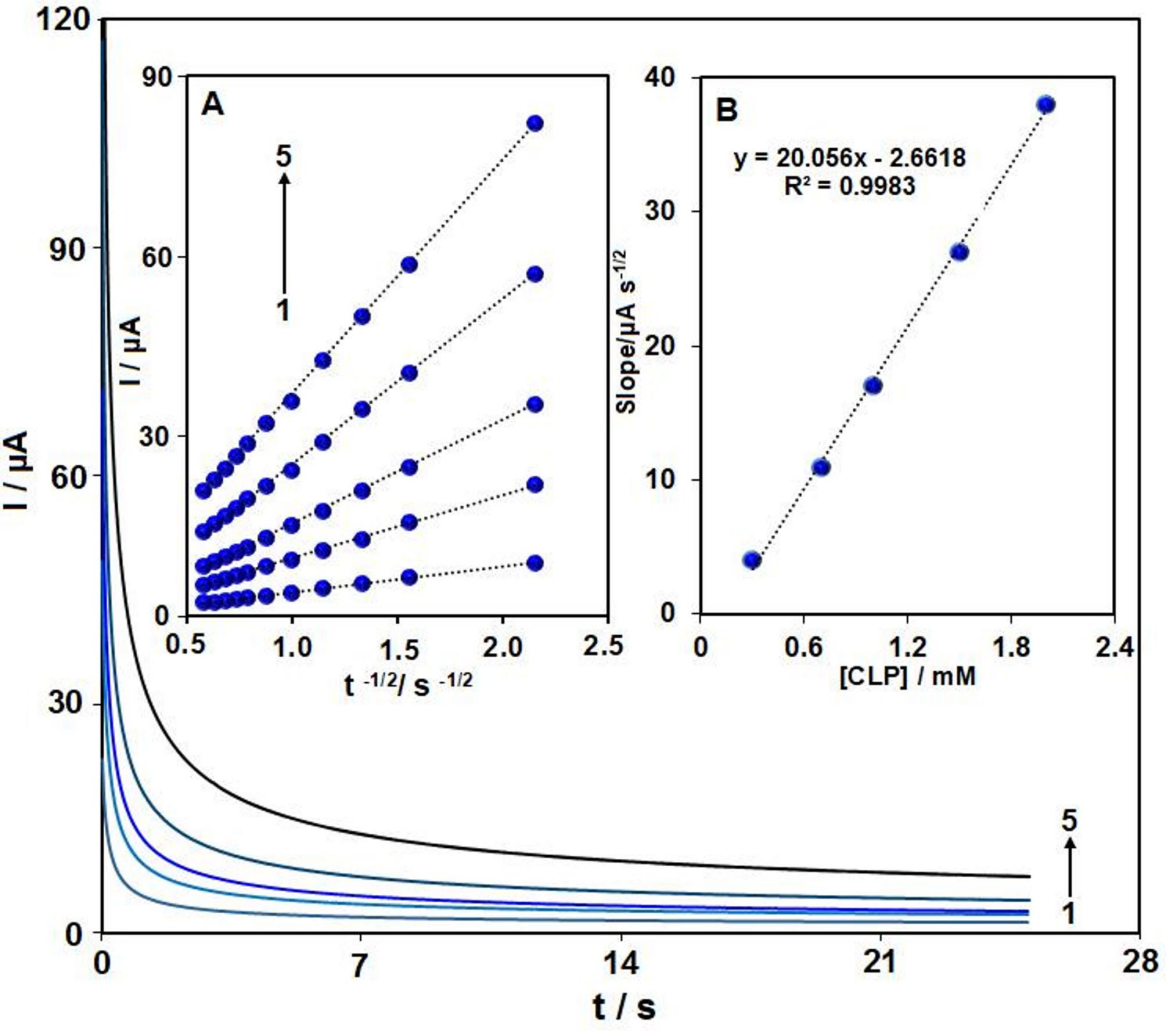
Chronoamperograms for Pr–CeO_2_–Bi_2_O_3_/IL/CPE in PBS (0.1 M, pH 7) for various concentrations of CLP (values 1–5 corresponding to 0.3, 0.7, 1.0, 1.5 and 2.0 mM CLP, respectively). Insets: (**A**) I vs. t^− 1/2^ plots derived from chronoamperograms 1–5. (**B**) The slopes of the straight lines versus the concentrations of CLP.

Chronoamperometric analysis of chlorpromazine at various concentrations was performed using Pr–CeO_2_–Bi_2_O_3_/IL/CPE at a potential of 640 mV in PBS (pH 7.0) (Fig. 7). The chronoamperometric response for electroactive species under mass transfer-limited conditions follows the Cottrell equation (I = n F A C_b_ D^1/2^ π^−1/2^ t^− 1/2^).

The Fig. 7-inset displays the plot of current (I) versus different CLP concentrations, with the data fitting optimally to straight lines. The slopes of these straight lines, as shown in inset B, were correlated with the CLP concentration. By applying the Cottrell equation and the obtained slopes, the average diffusion coefficient for CLP was calculated to be 1.47 × 10^–5^ cm^2^/s.

### Determination of CLP

**Fig. 8:**
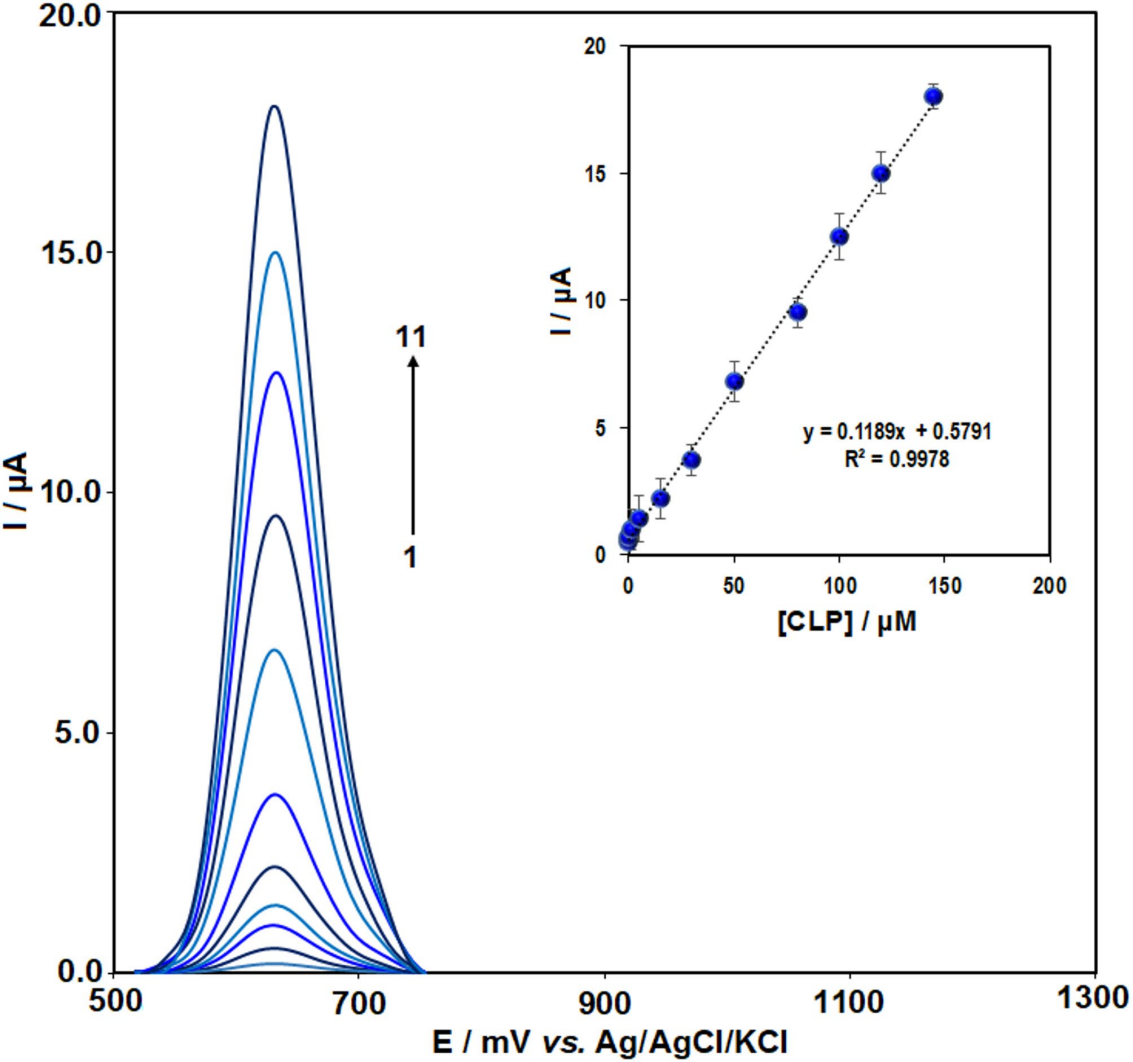
The differential pulse voltammetry responses for Pr–CeO_2_–Bi_2_O_3_/IL/CPE by adding CLP in a concentration range of 0.02 to 140 µM in PBS (0.1 M, pH 7). Inset: The relationship between the peak current and the concentration of CLP (*n* = 3).

The determination of chlorpromazine was performed using DPV at the Pr–CeO_2_–Bi_2_O_3_/IL/CPE in PBS (0.1 M, pH 7.0). As depicted in Fig. 8, the oxidation current (I_p_) rises progressively with increasing CLP concentration, demonstrating a linear correlation between I_p_ and CLP concentration over the range of 0.02 to 140 µM. The linear equation was y = 0.1189x + 0.5791 (R^2^ = 0.997).

Using the formula of LOD = S_b_/m, the LOD value of 0.009 µM was determined. The linear range and detection limits for CLP were compared with the results of other reported modified electrodes, and the findings are summarized in Table 1. The present method shows a broader linear working range and lower LOD than other methods. Additionally, this approach offers several advantages, including simple and rapid electrode preparation, minimal use of organic solvents, low cost, sufficient sensitivity, and good repeatability, making it suitable for real sample applications.

**Table 1:** Comparative analysis of analytical parameters for chlorpromazine detection using Pr–CeO_2_–Bi_2_O_3_/IL/CPE and other reported sensors.

Electrode	Linear range (µM)	LOD (nM)	Ref.
Au/Cu nanoparticles	0.1–1000	70	^46^
Zn–Al LDH	100–1000	16	^47^
fluorine-doped tin oxide (FTO) electrode	2-100	260	^48^
MOF@g-C_3_N_4_	0.02–2.99	2.45	^49^
Ni-MOF/Fe-MOF-5	0.001–900	25	^50^
SPO/PPy/SPCE	0.8–1207	69	^51^
FeVO_4_ NPs	0.04 -2222.1	4	^52^
Cys-CPE	1–35	11.9	^53^
SOD-GF/GCE	0.5–29.5	20	^54^
SrM/SPCE	0.1–143 and 153–1683	28	^55^
MWCNTs/PDA/AuNPs/ANE	100–450	22	^56^
Co-TMPA/MWCNT-2@GCE	0.05 to 1350	8	^57^
Pr-CeO_2_- Bi_2_O_3_/IL/CPE	0.02–140	9	This study

### Reproducibility, repeatability, and storage stability of the modified electrode

To evaluate the reproducibility of the Pr–CeO_2_–Bi_2_O_3_/IL/CPE, three sensors were prepared in the same manner and used to detect a 50 µM CLP solution. The RSD% was calculated to be 3.9% (*n* = 5), demonstrating the acceptable reproducibility of the proposed electrode. Furthermore, the repeatability of the proposed sensor was assessed by successively determining the 50 µM CLP solution eight times using the Pr–CeO_2_–Bi_2_O_3_/IL/CPE. The RSD value was 2.5%, confirming the suitable repeatability of the sensor. Subsequently, the storage stability of the electrode was also evaluated. After being stored at ambient temperature for three weeks, the electrode retained 92% of its initial current response, indicating good stability over time.

### Selectivity of the proposed sensor

**Fig. 9:**
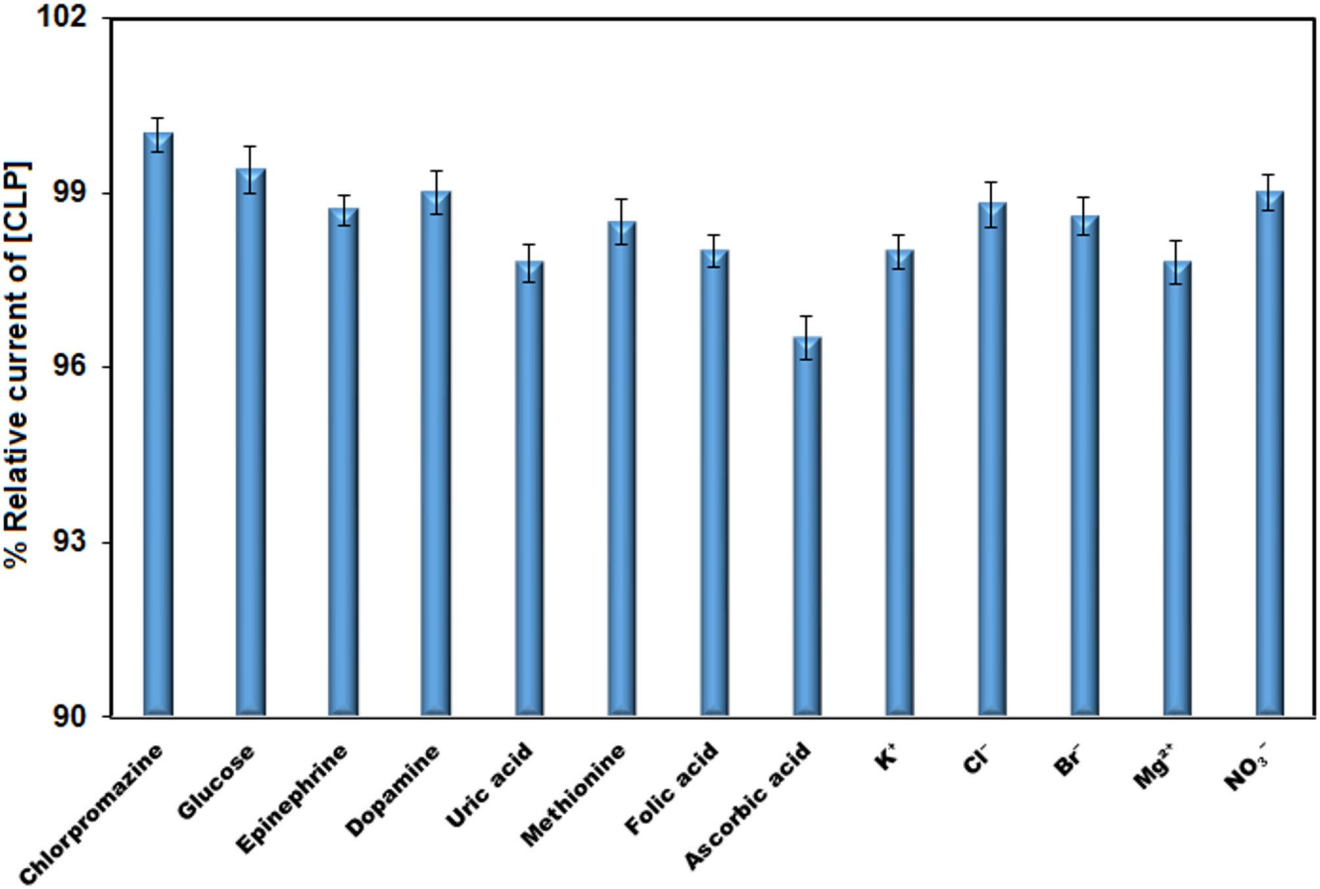
The diagram illustrating the impact of several interfering compounds on the response of 10 µM chlorpromazine (*n* = 3).

The selectivity of Pr–CeO_2_–Bi_2_O_3_/IL/CPE was evaluated by adding 50 times the concentration of various ions (K^+^, Mg^2+^, Br^−^, NO_3_^−^, and Cl^−^) to a solution containing 10 µM CLP. The presence of these inorganic ions did not interfere with the CLP detection, as the peak current changes were all less than ± 5%. Additionally, when the concentration of other potential interfering substances including ascorbic acid, epinephrine, glucose, uric acid, methionine, folic acid, and dopamine, was increased to 100 times that of CLP, the anodic current changes were still less than 5% (Fig. 9). These findings demonstrate that the modified electrode exhibits excellent selectivity for CLP, with minimal interference from various ions and other common substances, making it suitable for detecting the drug in complex real samples.

### Detection of real samples

The proposed modified electrode was employed to determine CLP in different real samples via DPV and the standard addition method. Prior to the real sample analysis, the stock solution was prepared, and known levels of the CLP were spiked into the real samples. As shown in Table 2, the recoveries of CLP are between 97.3% and 104% and RSDs are calculated to be lower than 4%. The results show that Pr–CeO_2_– Bi_2_O_3_/IL/CPE sensor can effectively detect CLP in real samples with acceptable accuracy and precision. The outputs acquired from the electrochemical measurements were compared with those from the HPLC method using the t-test statistical analysis. Since the calculated t-values were below the theoretical values at a 95% confidence level, no significant differences were observed between the methods (Table 3). These findings indicate that the proposed method does not significantly differ in accuracy from the reference method.

**Table 2:** Determination of chlorpromazine (CLP) in real samples using the proposed DPV method.

Sample	Spiked (µM)	Found (µM)	Recovery (%)	RSD (%)
Tablet	0.0	5.0	–	4.0
	5.0	9.8	98.0	2.5
	10.0	14.6	97.3	3.3
	15.0	20.8	104.0	3.4
Urine	0.0	N.D	–	–
	5.0	5.1	102.0	3.0
	8.0	7.8	97.5	2.1
	11.0	11.4	103.6	2.6

**Table 3:** Data validation through comparison with electrochemical and HPLC methods (*n* = 3).

Sample	Spiked (µM)	Found (µM)		t _exp_.
		DPV	HPLC	
Tablet	0.0	5.05 ± 0.41	5.02 ± 0.23	0.11
	5.0	9.83 ± 0.29	10.16 ± 0.36	1.23
Urine	5.0	5.09 ± 0.33	4.85 ± 0.18	1.10
	8.0	7.83 ± 0.28	8.16 ± 0.44	1.09

## Conclusion

In this study, Pr–CeO_2_–Bi_2_O_3_ nanoparticles were synthesized through a green and facile method employing L-alanine as both an environmentally benign reducing agent and a morphology-directing agent. A novel Pr–CeO_2_–Bi_2_O_3_/IL-modified electrode was then constructed for the electrochemical determination of CLP. The combination of Pr–CeO_2_–Bi_2_O_3_ nanostructures with the ionic liquid significantly enhanced conductivity, enlarged the electroactive surface area, and facilitated charge-transfer processes compared with the bare CPE. Owing to these synergistic effects, the sensor achieved several notable advances: (i) a wide linear range, (ii) an impressively low LOD of 0.009 µM, (iii) suitable reproducibility and repeatability, and (iv) good long-term stability. The sensor’s applicability in real sample analysis further highlighted its practical potential. Overall, this work provides not only a sensitive and reliable electrochemical sensing platform for CLP but also a sustainable and eco-friendly synthetic strategy for functional nanomaterials, underscoring the dual contribution of analytical performance and green chemistry.

## Data Availability

The datasets used and analysed during the current study are available from the corresponding author on reasonable request.

## References

[1] López-Muñoz, F. et al. History of the discovery and clinical introduction of chlorpromazine. *Ann. Clin. Psychiatry*. **17**, 113–135 (2005). 16433053 10.1080/10401230591002002

[2] Boyd-Kimball, D. et al. Classics in chemical neuroscience: chlorpromazine. *ACS Chem. Neurosci.* **10**, 79–88 (2018). 29929365 10.1021/acschemneuro.8b00258

[3] Meng, Q., Li, R., Hou, F. & Zhang, Q. Effects of chlorpromazine on sleep quality, clinical and emotional measures among patients with schizophrenia. *Clin. Neurol. Neurosurg.* **165**, 134–138 (2018). 29421173 10.1016/j.clineuro.2018.01.007

[4] McIver, B., Walsh, D. & Nelson, K. The use of chlorpromazine for symptom control in dying cancer patients. *J. Pain Symptom Manag.* **9**, 341–345 (1994). 10.1016/0885-3924(94)90193-7 7963786

[5] De Fazio, P. et al. Rare and very rare adverse effects of clozapine. *Neuropsychiatr. Dis. Treat.* 1995–2003 (2015). 10.2147/NDT.S83989 PMC4532211 26273202

[6] Saha, K. B. et al. Chlorpromazine versus atypical antipsychotic drugs for schizophrenia. *Cochrane Database Syst. Rev.* (2016). 10.1002/14651858.CD010631.pub2 PMC7081571 27045703

[7] Hillaert, S., Snoeck, L. & Van den Bossche, W. Optimization and validation of a capillary zone electrophoretic method for the simultaneous analysis of four atypical antipsychotics. *J. Chromatogr. A*. **1033**, 357–362 (2004). 15088758 10.1016/j.chroma.2004.01.057

[8] Mokhtari, A. & Rezaei, B. Chemiluminescence determination of chlorpromazine and fluphenazine in pharmaceuticals and human serum using Tris (1, 10-phenanthroline) ruthenium (II). *Anal. Methods*. **3**, 996–1002 (2011).

[9] Qi, L., Wang, Z., Chen, J. & Xie, J. W. Development and validation of a QuEChERS-HPLC-DAD method using polymer-functionalized melamine sponges for the analysis of antipsychotic drugs in milk. *Food Chem.* **444**, 138553 (2024). 38309075 10.1016/j.foodchem.2024.138553

[10] Kul, A. & Sagirli, O. A new method for the therapeutic drug monitoring of chlorpromazine in plasma by gas chromatography–mass spectrometry using dispersive liquid–liquid Microextraction. *Bioanalysis* **15**, 1343–1354 (2023). 37847049 10.4155/bio-2023-0176

[11] Koventhan, C., Shanmugam, R. & Chen, S. M. Development of highly sensitive electrochemical sensor for antipsychotic drug perphenazine using perovskite structured lanthanum Cobalt oxide nanoparticles wrapped graphitic carbon nitride nanocomposites. *Electrochim. Acta*. **467**, 143096 (2023).

[12] Koventhan, C., Kumar, N. K. R., Chen, S. M., Pandi, K. & Sangili, A. Polyol mediated synthesis of hexagonal manganese cobaltate nanoparticles for voltammetric determination of thioridazine. *Colloids Surf. Physicochem. Eng. Asp*. **621**, 126625 (2021).

[13] Maghsoodlou, M. T. et al. A facile synthesis of stable phosphorus ylides containing Chlorine and sulfur derived from 6-chloro-2-benzoxazolethiol and 2-chloro-phenothiazine. *Phosphorus Sulfur Silicon Relat. Elem* **184**, (2009).

[14] Liang, X., Zhou, Y., Almeida, J. M. S. & Brett, C. M. A. A novel electrochemical acetaminophen sensor based on multiwalled carbon nanotube and Poly (neutral red) modified electrodes with electropolymerization in ternary deep eutectic solvents. *J. Electroanal. Chem.* **936**, 117366 (2023).

[15] Mohammadnezhad, K., Ahour, F. & Keshipour, S. Electrochemical determination of ascorbic acid using palladium supported on N-doped graphene quantum Dot modified electrode. *Sci. Rep.* **14**, 5982 (2024). 38472243 10.1038/s41598-024-56231-x PMC10933321

[16] Tantawy, M. A., Wahba, I. A., Saad, S. S. & Ramadan, N. K. Two fabricated carbon paste electrodes for novel potentiometric determination of probenecid in dosage form and human plasma. *Sci. Rep.* **12**, 20418 (2022). 36443448 10.1038/s41598-022-24920-0 PMC9705367

[17] Moghaddam, H. M. Electrocatalytic determination of carbidopa and acetaminophen using a modified carbon nanotube paste electrode. *Int. J. Electrochem. Sci.* **6**, 6557–6566 (2011).

[18] Moghaddam, H. M., Malakootian, M., Beitollah, H. & Biparva, P. Nanostructured base electrochemical sensor for determination of sulfite. *Int. J. Electrochem. Sci.* **9**, 327–341 (2014).

[19] Moolayadukkam, S., Datta, P., Chowdhury, D. & Puri, I. K. Modifying metal ion ratios in nickel-aluminum layered double hydroxide/reduced graphene oxide composites for selective electrochemical detection of antibiotics. *Results Eng.* **24**, 103425 (2024).

[20] Ahmed, Y. M., Eldin, M. A., Galal, A. & Atta, N. F. Electrochemical sensor based on PEDOT/CNTs-graphene oxide for simultaneous determination of hazardous hydroquinone, catechol, and nitrite in real water samples. *Sci. Rep.* **14**, 5654 (2024). 38454022 10.1038/s41598-024-54683-9 PMC10920748

[21] Malakootian, M., Hamzeh, S. & Mahmoudi-Moghaddam, H. A. Novel electrochemical sensor based on FeNi3/CuS/ BiOCl modified carbon paste electrode for determination of bisphenol A. *Electroanalysis* **33**, 38–45 (2021).

[22] Moghaddam, H. M., Beitollahi, H., Dehghannoudeh, G. & Forootanfar, H. A label-free electrochemical biosensor based on carbon paste electrode modified with graphene and ds-DNA for the determination of the anti-cancer drug Tamoxifen. *J. Electrochem. Soc.* **164**, B372 (2017).

[23] Koventhan, C., Pandiyan, R. & Chen, S. M. Rational design of dysprosium Cobalt oxide decorated on flower-like molybdenum disulfide: development of an electrochemical sensor for antipsychotic drug promazine. *Process Saf. Environ. Prot.* **170**, 1188–1199 (2023).

[24] Koventhan, C., Pandiyan, R., Chen, S. M. & Lo, A. Y. Nickel molybdate/cobalt molybdate nanoflakes by one-pot synthesis approach for electrochemical detection of antipsychotic drug chlorpromazine in biological and environmental samples. *J. Environ. Chem. Eng.* **11**, 110121 (2023).

[25] Pitiphattharabun, S. et al. Reduced graphene oxide/zinc oxide composite as an electrochemical sensor for acetylcholine detection. *Sci. Rep.* **14**, 14224 (2024). 38902301 10.1038/s41598-024-64238-7 PMC11190213

[26] Moghaddam, H. M. & Malakootian, M. Differential pulse voltammetric determination of Levodopa in pharmaceutical and biological samples using nio/graphene oxide nanocomposite modified graphite screen printed electrode. *Anal. Bioanal. Electrochem.* **10**, 520–530 (2018).

[27] Wang, Y. et al. Stripping voltammetric determination of cadmium and lead ions based on a bismuth oxide surface-decorated nanoporous bismuth electrode. *Electrochem. Commun.* **136**, 107233 (2022).

[28] Kokulnathan, T., Vishnuraj, R., Wang, T. J., Kumar, E. A. & Pullithadathil, B. Heterostructured bismuth oxide/hexagonal-boron nitride nanocomposite: A disposable electrochemical sensor for detection of Flutamide. *Ecotoxicol. Environ. Saf.* **207**, 111276 (2021). 32931965 10.1016/j.ecoenv.2020.111276

[29] Sivasubramanian, P. et al. A review on bismuth-based nanocomposites for energy and environmental applications. *Chemosphere* **307**, 135652 (2022). 35817189 10.1016/j.chemosphere.2022.135652

[30] Hossain, M. K., Ahmed, M. H., Khan, M. I., Miah, M. S. & Hossain, S. Recent progress of rare Earth oxides for sensor, detector, and electronic device applications: a review. *ACS Appl. Electron. Mater.* **3**, 4255–4283 (2021).

[31] Khan, S. U., Khalid, W., Atif, M. & Ali, Z. Graphene oxide–cerium oxide nanocomposite modified gold electrode for ultrasensitive detection of Chlorpyrifos pesticide. *RSC Adv.* **14**, 27862–27872 (2024). 39224650 10.1039/d4ra04406a PMC11367620

[32] Kim, S. et al. A novel electrochemical strategy to detect hydrogen peroxide by utilizing peroxidase-mimicking activity of cerium oxide/graphene oxide nanocomposites. *Biosens. Bioelectron.* **253**, 116161 (2024). 38457864 10.1016/j.bios.2024.116161

[33] Mahmoudi-Moghaddam, H., Javar, A., Garkani-Nejad, Z. & H. & Fabrication of platinum-doped NiCo2O4 Nanograss modified electrode for determination of carbendazim. *Food Chem.* **383**, 132398 (2022). 35183970 10.1016/j.foodchem.2022.132398

[34] Sardar, K. et al. Nanocrystalline cerium–bismuth oxides: synthesis, structural characterization, and redox properties. *Chem. Mater.* **22**, 6191–6201 (2010).

[35] Sudzhanskaya, I. V. & Sotnikova, V. S. Electrical characteristics of Bismuth-Containing cerium oxide. *Glass Ceram.* **80**, 100–104 (2023).

[36] Al-Sodies, S., Asiri, A. M., Khan, A., Alamry, K. A. & Hussein, M. A. Recent exploiting of Poly (ionic liquid) s in sensing applications. *Eur. Polym. J.* **112719** (2023).

[37] Thakur, A. & Kumar, A. Exploring the potential of ionic liquid-based electrochemical biosensors for real-time biomolecule monitoring in pharmaceutical applications: from lab to life. *Results Eng.* **20**, 101533 (2023).

[38] Opallo, M. & Lesniewski, A. A review on electrodes modified with ionic liquids. *J. Electroanal. Chem.* **656**, 2–16 (2011).

[39] Khan, A. B. Green chemistry of ionic liquids in surface electrochemistry. In *Advanced Applications of Ionic Liquids*, 89–111 (Elsevier, 2023).

[40] Kumar, J. A. et al. A focus to green synthesis of metal/metal based oxide nanoparticles: various mechanisms and applications towards ecological approach. *J. Clean. Prod.* **324**, 129198 (2021).

[41] Ali, H. H. et al. Wet-chemical Preparation and physicochemical characterization of nanostructured Zn-Bi2O3/CeO2 composite: A visible-light-driven catalyst for the annihilation of pharmaceutical pollutant. *Ceram. Int.* **50**, 52572–52582 (2024).

[42] Mahdavi, K., Zinatloo-Ajabshir, S., Yousif, Q. A. & Salavati-Niasari, M. Enhanced photocatalytic degradation of toxic contaminants using Dy2O3-SiO2 ceramic nanostructured materials fabricated by a new, simple and rapid sonochemical approach. *Ultrason. Sonochem*. **82**, 105892 (2022). 34959201 10.1016/j.ultsonch.2021.105892 PMC8799595

[43] Valian, M., Mahdi, M. A., Jasim, L. S. & Salavati-Niasari, M. Significantly improved electrochemical hydrogen storage accomplishment promoted by novel Li0. 35La0. 55TiO3/rGO nanocomposite. *J. Energy Storage*. **84**, 110862 (2024).

[44] Heydariyan, Z. et al. Facile Preparation and characterization of SmMn2O5/Mn2O3/g-C3N4 nanocomposites for electrochemical hydrogen storage application. *Int. J. Hydrogen Energy*. **90**, 1300–1312 (2024).

[45] Heidari-Asil, S. A., Zinatloo-Ajabshir, S., Amiri, O. & Salavati-Niasari, M. Amino acid assisted-synthesis and characterization of magnetically retrievable ZnCo2O4–Co3O4 nanostructures as high activity visible-light-driven photocatalyst. *Int. J. Hydrogen Energy*. **45**, 22761–22774 (2020).

[46] Chen, J. et al. An electrochemical chlorpromazine sensor based on a gold–copper bimetallic synergetic molecularly imprinted interface on an acupuncture needle electrode. *Analyst* **148**, 2214–2224 (2023). 37114554 10.1039/d3an00373f

[47] Srinivasan, S. et al. Electrochemical sensor based on electrodeposited Zinc-Aluminium layered double hydroxide modified glassy carbon electrode for chlorpromazine sensing. *J. Electrochem. Soc.* **171**, 037517 (2024).

[48] Martinez-Rojas, F., Espinosa-Bustos, C., Ramirez, G. & Armijo, F. Electrochemical oxidation of chlorpromazine, characterisation of products by mass spectroscopy and determination in pharmaceutical samples. *Electrochim. Acta*. **443**, 141873 (2023).

[49] Ashkar, M. A. et al. Design of sonochemical assisted synthesis of Zr-MOF/g-C3N4-modified electrode for ultrasensitive detection of antipsychotic drug chlorpromazine from biological samples. *Microchim. Acta*. **191**, 182 (2024). 10.1007/s00604-024-06253-z 38451377

[50] Lu, Z. et al. Nanoarchitectonics of on–off ratiometric signal amplified electrochemical sensor for chlorpromazine with molecularly imprinted polymer based on Ni-MOF/Fe-MOF-5 hybrid Au nanoparticles. *Sep. Purif. Technol.* **327**, 124858 (2023).

[51] Vinothkumar, V., Sakthivel, R., Chen, S. M., Abinaya, M. & Kubendhiran, S. Facile synthesis of alpha-phase strontium pyrophosphate incorporated with polypyrrole composite for the electrochemical detection of antipsychotic drug chlorpromazine. *J. Alloys Compd.* **888**, 161537 (2021).

[52] Kesavan, G., Gopi, P. K., Chen, S. M. & Vinothkumar, V. Iron vanadate nanoparticles supported on Boron nitride nanocomposite: electrochemical detection of antipsychotic drug chlorpromazine. *J. Electroanal. Chem.* **882**, 114982 (2021).

[53] Purushothama, H. T. et al. Electrochemical determination of chlorpromazine using l-Cysteine modified carbon paste electrode. *Chem. Data Collections*. **23**, 100268 (2019).

[54] Parfait Tchoumi, F. et al. Electrochemical behaviour and sensing of chlorpromazine at polymer-free kaolin‐based nanosodalite and nanosodalite‐graphene foam film modified glassy carbon electrodes. *ChemElectroChem*. e202400080 (2024).

[55] Kumar, J. V. et al. Highly selective electrochemical detection of antipsychotic drug chlorpromazine in drug and human urine samples based on peas-like strontium molybdate as an electrocatalyst. *Inorg. Chem. Front.* **5**, 643–655 (2018).

[56] Zhan, S., Liu, H., Li, L., Yin, Z. & Gu, C. Simultaneously determining dopamine, acetaminophen and chlorpromazine with high performance on coordinated functional acupuncture needle electrode. *Microchem. J.* **204**, 111085 (2024).

[57] Ma, L., Pei, W. Y., Yang, J. & Ma, J. F. Efficient electrochemical sensing of chlorpromazine with a composite of multiwalled carbon nanotubes and a Thiacalix[4]arene-Based Metal–Organic framework. *Langmuir* **40**, 17656–17666 (2024). 39161301 10.1021/acs.langmuir.4c02003

